# Prediction of IOL decentration, tilt and axial position using anterior segment OCT data

**DOI:** 10.1007/s00417-023-06208-9

**Published:** 2023-09-01

**Authors:** Achim Langenbucher, Nóra Szentmáry, Alan Cayless, Jascha Wendelstein, Peter Hoffmann

**Affiliations:** 1 Department of Experimental Ophthalmology, Saarland University, /Saar, 66424 Homburg, Germany; 2 Dr. Rolf M. Schwiete Center for Limbal Stem Cell and Aniridia Research, Saarland University, /Saar, 66424 Homburg, Germany; 3 Department of Ophthalmology, Semmelweis-University, Budapest, Hungary; 4 School of Physical Sciences, The Open University, Milton Keynes, United Kingdom; 5 Department of Ophthalmology, Johannes Kepler University Linz, Linz, Austria; 6 Augen- und Laserklinik Castrop-Rauxel, Castrop-Rauxel, Germany

**Keywords:** IOL tilt, IOL decentration, Effective IOL position, Geometric IOL position, Anterior segment tomographer, Prediction model, Feedforward neural network, Multilinear regression model

## Abstract

**Background:**

Intraocular lenses (IOLs) require proper positioning in the eye to provide good imaging performance. This is especially important for premium IOLs. The purpose of this study was to develop prediction models for estimating IOL decentration, tilt and the axial IOL equator position (IOLEQ) based on preoperative biometric and tomographic measures.

**Methods:**

Based on a dataset (*N* = 250) containing preoperative IOLMaster 700 and pre-/postoperative Casia2 measurements from a cataractous population, we implemented shallow feedforward neural networks and multilinear regression models to predict the IOL decentration, tilt and IOLEQ from the preoperative biometric and tomography measures. After identifying the relevant predictors using a stepwise linear regression approach and training of the models (150 training and 50 validation data points), the performance was evaluated using an *N* = 50 subset of test data.

**Results:**

In general, all models performed well. Prediction of IOL decentration shows the lowest performance, whereas prediction of IOL tilt and especially IOLEQ showed superior performance. According to the 95% confidence intervals, decentration/tilt/IOLEQ could be predicted within 0.3 mm/1.5°/0.3 mm. The neural network performed slightly better compared to the regression, but without significance for decentration and tilt.

**Conclusion:**

Neural network or linear regression-based prediction models for IOL decentration, tilt and axial lens position could be used for modern IOL power calculation schemes dealing with ‘real’ IOL positions and for indications for premium lenses, for which misplacement is known to induce photic effects and image distortion.

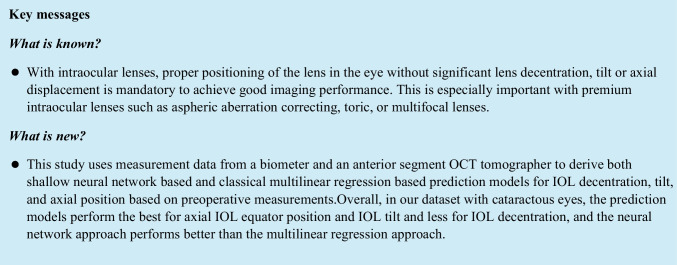

## Introduction

Prediction of the pseudophakic intraocular lens (IOL) position is one of the remaining challenges in modern cataract surgery. All theoretical-optical lens formulae either implicitly or explicitly require a prediction of the axial IOL position, as do numerical raytracing methods based on pseudophakic model eyes [1–3]. The classical formulae, in particular, deal with an effective lens position (ELP). This, however, does not coincide with the geometrical axial IOL position in the pseudophakic eye, mostly due to assumptions on the conversion of corneal front surface radius to corneal power. However, modern formulae or numerical raytracing mostly deal with the ‘anatomically correct’ axial lens position (ALP), and this has to be predicted during the biometry and IOL power calculation before cataract surgery [4, 5].

In addition to the axial IOL position, the lateral displacement (decentration in *X* and *Y* direction) or the tilt (around *X* and *Y*) is also known to be relevant determinants for image performance after cataract surgery. This is particularly important in the context of premium IOL implantation [6–11]. Since the visual axis is typically slanted with respect to the ‘optical axis’ or ‘symmetry axis’ because of the eccentric location of the fovea, the optical elements are not properly aligned to the visual axis [12]. This means that rays passing through the pupil of the eye towards the fovea hit the cornea somewhat nasally, and both the cornea and the IOL are somewhat tilted with respect to the visual axis. For example, in the Liou-Brennan schematic model eye [13], as mostly used for modern raytracing purposes, the pupil is shifted by half a millimetre and the entrance beam is slanted by 5° in the nasal direction if the refractive elements of the cornea and lens are aligned to the ‘optical axis’.

IOL decentration and tilt relative to the visual axis are known to induce some astigmatism as well as asymmetric higher-order aberrations in the wavefront. These can deteriorate the imaging properties of the eye [1, 7, 11]. Particularly in the premium IOL segment with refractive or diffractive bi- or multifocal IOLs, the efficiency of the near-addition segments can be significantly affected. With toric IOLs, in addition to decentration and tilt, rotation around the *Z* axis (IOL rotation) affects the astigmatic correction. Even slight rotations of a fully correcting toric IOL reduce the efficiency of astigmatic correction by 15%, and a rotation of 30° shows no more astigmatic correction effect and simply turns the axis of the resulting cylinder. Decentration of optical elements in the eye with respect to the visual axis cannot be considered using formula-based IOL calculations, but modern raytracing techniques allow both the decentration and tilt of the IOL, and the pupil outline, to be included in the calculation, enabling the visual performance of the pseudophakic eye to be predicted with various lens designs.

The prediction of the IOL position seems to be a ‘Holy Grail’ of theoretical-optical lens power formulae or raytracing [2, 5]. An over-estimation/under-estimation makes the patient’s eye (treated with an IOL with plus power) myopic/hyperopic. The amount of error refraction depends strongly on the biometric measures and the power of the implanted IOL, and with a ‘normal’ eye, a 1-mm prediction error in the axial IOL position shifts the refraction at the spectacle plane by around 1.6 dpt.

Classical imaging techniques such as Purkinje image evaluation could be used to obtain a rough estimate for the tilt angle between the visual axis and the optical axis of the eye. Analysing the corneal reflex position (Purkinje image I) with respect to the centre of the entrance pupil (the so-called chord mu or CW chord) allows the angle alpha/kappa or the displacement of the corneal centre from the chief ray through the eye to be evaluated. Analysis of Purkinje images III and IV with respect to Purkinje I gives some insight into lens decentration and tilt, but quantification might be challenging. In most cases, the analysis of Purkinje images is performed qualitatively. Since the light reflexes of the corneal back surfaces and the lens front and back surface have to pass through the refractive surfaces located further forward, an inverse raytracing procedure is required to determine decentration and tilt of the lens from Purkinje images III and IV, and in most cases, a simple linear approximation is used instead [14]. However, general guidelines for cataract surgery with premium IOLs include measurement of chord mu before surgery, and indication for multifocal or EDOF IOLs should be carefully considered with large values of chord mu.

Modern optical measurement techniques such as high-resolution anterior segment optical coherence tomographers (OCT) are capable of measuring the axial position (front and back apex position), decentration and tilt of the crystalline lens/IOL before/after cataract surgery [12]. With a pupil size of around 3 mm or more, novel plug-in software tools (e.g. lens analysis module) can fit the crystalline lens front and back surface. For example, with a sphere and by extrapolation of both fit surfaces, the equatorial plane can be determined, together with an estimate for the equatorial lens diametre [12]. With this lens analysis technique, the crystalline lens is split into an anterior portion (from the front apex to the equator plane) and a posterior portion (from the equator to the posterior apex).

The purpose of this study was

- to use anterior segment measurements from the Casia2 optical coherence tomographer to quantify lateral decentration, tilt and axial position of the crystalline lens front and back vertex and equator plane,
- to measure lateral decentration, tilt and axial position of the IOL front and back vertex,
- to set up feedforward neural network and multilinear regression-based prediction models to estimate the postoperative decentration, tilt and axial position based on preoperative biometric measures, and
- to analyse the prediction results on a dataset with clinical data from 250 cataract surgeries.

## Materials and methods

### Dataset for analysis

In this retrospective study, we analysed a dataset containing measurements from 250 eyes from a cataract population from the Augen- und Laserklinik Castrop-Rauxel, Castrop-Rauxel, Germany which was transferred to us. In the dataset, only one eye for each patient was included, and eyes with zonule weakness (e.g. pseudoexfoliation syndrome) or with any history of ocular surgery were excluded before the dataset was transferred to us. The dataset contains patient age, sex, eye side, preoperative biometry measurements with the IOLMaster 700 (IOLM, Carl-Zeiss-Meditec AG, Jena, Germany), preoperative and 4–12 weeks postoperative Casia2 (CASIA, Tomey GmbH, Nürnberg, Germany, software version Ver.50.5A.03) anterior segment OCT measurements including lens analysis protocol, and postoperative (4-12 weeks postoperatively) refraction data. The raw data (.XLSX-format) were transferred to us in an anonymised fashion, precluding back-tracing of the patient. The XLSX data were imported into MATLAB (Matlab 2021a, MathWorks, Natick, USA) for further processing.

### Preprocessing of the data

Custom software was written in Matlab. The dataset included the following.

Preoperative IOLM measurement: Axial length measurement (AL in mm), central corneal thickness (CCT in mm), external anterior chamber depth measured from corneal epithelium to lens front apex (ACD in mm), lens thickness (LT in mm), horizontal corneal diameter (CD in mm) and radius of curvature of the corneal front surface (R1/R2 in mm in the flat/steep corneal meridian with axis R1A and R2A in °).

Preoperative (._pr_) and postoperative (._po_) CASIA: central corneal thickness (CCT_pr_ and CCT_po_ in mm), external anterior chamber depth (ACD_pr_ and ACD_po_ in mm), lens thickness (LT_pr_ and LT_po_ in mm), horizontal corneal diameter (CD_pr_ and CD_po_ in mm) and radius of curvature of the corneal front surface (R1_pr_/R2_pr_ and R1_po_/R2_po_ in mm in the flat/steep corneal meridian with axis R1A_pr_/R2A_pr_ and R1A_po_/R2A_po_ in °) and back surface (R1b_pr_/R2b_pr_ and R1b_po_/R2b_po_ in mm in the flat/steep corneal meridian with axis R1b_pr_A/R2b_pr_A and R1b_po_A/R2b_po_A in °). In addition, from the lens analysis module, we extracted axial position of the equatorial lens plane (LEQ_pr_ in mm), equatorial diameter (LED_pr_ in mm), lens decentration (LDEC_pr_ and LDEC_po_ in mm with respective axes LDEC_pr_A and LDEC_po_A in °) and lens tilt (LTILT_pr_ and LTILT_po_ in ° with respective orientation axis LTILT_pr_A and LTILT_po_A in °).

The mean corneal radius of curvature was calculated from the preoperative and postoperative CASIA data for the front surface (R_pr_ = ½ (R1_pr_ + R2_pr_) and R_po_ = ½ (R1_po_ + R2_po_) and the back surface (Rb_pr_ = ½ (R1b_pr_ + R2b_pr_) and Rb_po_ = ½ (R1b_po_ + R2b_po_)). Preoperatively, the LEQ_pr_ value provided from the CASIA lens analysis software was quoted as axial equatorial lens position, and postoperatively LEQ_po_ was derived from half the distance between the IOL front and back apex (LEQ_po_ = ACD_po_ + ½ LT_po_). The absolute values of the preoperative and postoperative lens decentration and tilt were converted to component notation using the following:

$${\displaystyle \begin{array}{cc}\begin{array}{ccc}{LDEC}_{pr}X& =& {LDEC}_{pr}\bullet \cos \left({LDEC}_{pr}A\right)\\ {}{LDEC}_{pr}Y& =& {LDEC}_{pr}\bullet \sin \left({LDEC}_{pr}A\right)\end{array}& \begin{array}{ccc}{LDEC}_{po}X& =& {LDEC}_{po}\bullet \cos \left({LDEC}_{po}A\right)\\ {}{LDEC}_{po}Y& =& {LDEC}_{po}\bullet \sin \left({LDEC}_{po}A\right)\end{array}\\ {}\begin{array}{ccc}{LTILT}_{pr}X& =& {LTILT}_{pr}\bullet \cos \left({LTILT}_{pr}A\right)\\ {}{LTILT}_{pr}Y& =& {LTILT}_{pr}\bullet \sin \left({LTILT}_{pr}A\right)\end{array}& \begin{array}{ccc}{LTILT}_{po}X& =& {LTILT}_{po}\bullet \cos \left({LTILT}_{po}A\right)\\ {}{LTILT}_{po}Y& =& {LTILT}_{po}\bullet \sin \left({LTILT}_{po}A\right)\end{array}\end{array}}$$

Assuming symmetry between left and right eyes, all *X* components of lens decentration and tilt (LDEC_pr_X, LDEC_po_X, LTILT_pr_X and LTILT_po_X) were reversed in the sign for left eyes (OS) to consider all eyes in the study as right eyes (OD).

### Identification of potential predictors

Before setting up prediction models for postoperative IOL decentration and tilt and axial position of the IOL equator plane, the relevant parameters have to be identified. We used a stepwise linear regression approach [15], starting with a constant model and a set of potential predictors AL, CCT_pr_, ACD_pr_, LT_pr_, CD_pr_, LEQ_pr_ and the vector components LDEC_pr_X/LDEC_pr_Y, and LTILT_pr_X/LTILT_pr_Y, to predict the output parameters LDEC_po_X/LDEC_po_Y, and LTILT_po_X/LTILT_po_Y and LEQ_po_. In this stepwise linear regression approach, parameters are iteratively added or removed based on their impact on the model performance (the significance level for being entered/removed from regression was set to 0.01/0.1). For simplicity, we restricted the model to a multilinear model including intercept, but without interaction of parameters [15].

### Setup of the feedforward shallow neural network for prediction

A feedforward shallow multi-layer multi-output neural network (FFNN) [16, 17] was set up for predicting (A) IOL decentration (LDEC_po_X/LDEC_po_Y), (B) IOL tilt (LTILT_po_X/LTILT_po_Y) and (C) axial position of the IOL equator plane LEQ_po_. The parameters identified in the previous step were used as input parameters (predictors). The Levenberg-Marquardt algorithm [18, 19] was used as the training function, as this algorithm is known to have a good performance in terms of convergence and stability. Based on the squared prediction error (derived from the 2 component vectors for A and B or the scalar for C) (difference of predicted and observed/measured target value)

$${\displaystyle \begin{array}{c}A: sPE=1/2\bullet \left(\left({\textrm{predLDEC}}_{po}\textrm{X}-{\textrm{LDEC}}_{po}\textrm{X}\right)2+{\left({\textrm{predLDEC}}_{po}\textrm{Y}-{\textrm{LDEC}}_{po}\textrm{Y}\right)}^{{}^2}\right)\\ {}B: sPE=1/2\bullet \left({\left({\textrm{predLDEC}}_{po}\textrm{X}-{\textrm{LDEC}}_{po}\textrm{X}\right)}^{{}^2}+{\left({\textrm{predLDEC}}_{po}\textrm{Y}-{\textrm{LDEC}}_{po}\textrm{Y}\right)}^{{}^2}\right)\\ {}C: sPE={\left( predLEQpo- LEQpo\right)}^2\end{array}}$$

the optimisation was performed in terms of minimising the mean squared prediction error, which refers to a metric for the performance of the prediction [20]:

$$msPE=\frac{\sum_N sPE}{N}.$$

To keep the network structure simple, we decided to set up an FFNN with 2 hidden layers and 10/8 neurons in the first/second hidden layer [17, 19].

In the next step, the dataset with *N* = 250 data points was split using a random selection into a training set (60%, *N* = 150), a validation set (20%, *N* = 50) and a test set (20%, *N* = 50) [17]. The FFNN was trained using the training dataset and back-propagated with the validation dataset. The final proof was performed using the *N* = 50 subset of test data. In the final step, we used the FFNN to make a prediction for the entire dataset (*N* = 250 data points).

### Setup of the multilinear regression for prediction

Furthermore, we defined a multilinear linear regression model (MLRM) using the same input variables (predictors) as the FFNN to estimate IOL decentration (A), IOL tilt (B) and the axial position of the IOL equator plane (C) [15, 21]. To compare the performance of the FFNN and MLRM prediction, we used the training data from the previous step to train the MLRM and the test data to assess the performance of the prediction. Again, the squared prediction error was used as a quality metric. In the final step, we used the MLRM to make a prediction for the entire dataset (*N* = 250 data points).

### Statistical evaluation

Values listed in the data tables included the arithmetic mean, standard deviation, median and the lower and upper boundary of the 95% confidence interval (2.5% and 97.5% quantiles). The measured lens decentration and tilt before (crystalline lens) and after cataract surgery (IOL) and the prediction error of the prediction models are shown with scatterplots, in which the *X* component refers to the horizontal direction and the *Y* component to the vertical direction. The axial position of the lens equator plane before and after cataract surgery and the prediction error is displayed with Bland-Altman plots and histograms.

The performance of the FFNN and MLRM for the prediction of decentration and tilt are compared using a test strategy for multivariate testing. Depending on the normality of the data investigated with the Henze-Zirkler test for normality [22, 23], either a Hotelling-T^2^ test [24] or a multivariate rank sign test [25, 26] is used for normally distributed or non-normal data respectively. The performance of the FFNN and MLRM for the prediction of axial IOL equator position is compared using a test strategy for univariate testing. Depending on the normality of the data as assessed using a Shapiro-Wilk test for normality [27], either a *t*-test or a nonparametric Wilcoxon test for paired samples is used for normally distributed or non-normal data, respectively.

## Results

The dataset transferred to us contained *N* = 250 data points (126 eyes from female and 124 eyes from male patients, 128 right and 122 left eyes). The mean age was 70.74 ± 9.81 years (median 72.00 years, 95% CI 49.45 to 85.28 years). All data had already been checked for completeness at the source. In total, 4 types of lens were implanted: Alcon SA60AT (*N* = 24, Alcon, Fort Worth, USA), Johnson & Johnson Tecnis (*N* = 120, Johnson and Johnson, Brunswick, USA), Hoya Vivinex XC1 (*N* = 84, Hoya, Singapore) and Bausch & Lomb Envista MX60 (*N* = 22, Bausch and Lomb, Rochester, USA).

Table 1 displays the descriptive data for the most relevant biometric measures, including AL, ACD_pr_, LT_pr_, CD_pr_, R_pr_, Rb_pr_, LDEC_pr_X, LDEC_pr_Y, LTILT_pr_X, LTILT_pr_Y and LEQ_pr_. The power of the implanted lens was 20.7994 ± 4.0704 dpt (median 21.0 dpt; 95% CI 11.0 to 28.0 dpt), and the postoperative refraction at the spectacle plane (spherical equivalent power) was −0.5412 ± 0.8165 dpt (median −0.375; 95% CI −2.500 to 0.625 dpt).

**Table 1:** Descriptive data of preoperative measures

*N*= 250	AL in mm	CCT_pr_ in mm	ACD_pr_ in mm	LT_pr_ in mm	CD_pr_ in mm	R_pr_ in mm	LDEC_pr_X in mm	LDEC_pr_Y in mm	LTILT_pr_X in °	LTILT_pr_Y in °	LEQ_pr_ in mm
Mean	24.0071	0.5551	3.1742	4.5964	11.9430	7.7486	−0.1515	0.0387	−5.1868	−1.3401	4.7594
SD	1.6471	0.0354	0.4219	0.4180	0.4109	0.2575	0.1411	0.1321	1.5039	1.4253	0.3790
Median	23.7700	0.5540	3.2200	4.6000	12.0000	7.7400	−0.1513	0.0286	−5.1992	−1.6278	4.7650
2.5% quantile	21.5352	0.4917	2.3745	3.7735	11.1000	7.2322	−0.3897	−01858	−7.9460	−3.8097	4.0044
97.5% quantile	28.6210	0.6264	3.9428	5.4400	12.7275	8.4055	0.1418	0.3124	−2.0135	1.9576	5.4966

AL refers to axial length, CCT_pr_ to central corneal thickness, ACD_pr_ to anterior chamber depth, LT_pr_ to lens thickness, CD_pr_ to horizontal corneal diameter, R_pr_ and Rb_pr_ to the mean corneal radius for the front and back surface, LDEC_pr_X and LDEC_pr_Y to the decentration of the crystalline lens in *X* and *Y* directions, LTILT_pr_X and LTILT_pr_Y to the tilt of the crystalline lens around the *X* and *Y* axes, and LEQ_pr_ to the axial position of the equator plane of the crystalline lens. Data are given in terms of the mean, standard deviation (SD), median and the lower (2.5% quantile) and upper (97.5% quantile) boundaries of the 95% confidence interval

From the stepwise linear regression, we can see that for the prediction of IOL decentration LDEC_po_X/LDEC_po_Y that in addition to an intercept, we have to consider AL, LT_pr_ and LDEC_pr_X/LDEC_pr_Y as predictors. For the prediction of IOL tilt LTILT_po_X/LTILT_po_Y, in addition to the intercept, we have to consider AL, LT_pr_, LDEC_pr_X/LDEC_pr_Y and LTILT_pr_X/LTILT_pr_Y as predictors. For the prediction of the axial IOL equator position LEQ_po_, we identified AL, LEQ_pr_ and LDEC_pr_X as predictors in addition to the intercept. In the next step, these predictors were used to set up the feedforward neural network FFNN and multilinear regression model-based prediction for estimation of IOL decentration, tilt and axial IOL position.

In Table 2, we list the descriptive data of the performance characteristics for the feedforward neural network FFNN and for the multilinear regression model MLRM. All models were trained on the training set and tested on the test set. The validation set was used with the FFNN for backpropagation. On the left side, the mean squared prediction errors msPE of the FFNN are listed for the training data, validation data and test data, together with the optimal number of iteration cycles (epochs) during training (the msPE data refer to this iteration). On the right side, the mean squared prediction errors msPE of the MLRM are listed for the training data, validation data and test data, together with the coefficient of determination derived from the training set.

**Table 2:** Descriptive data of the performance characteristics for the feedforward neural network FFNN and the multilinear regression model MLRM.

Mean squared prediction error msPE (*N* = 250)	Feedforward neural network FFMM				Multilinear regression model MLRM			
	Training data (*N* = 150)	Validation data (*N* = 50)	Test data (*N* = 50)	Iterations (epochs)	Training data (*N* = 150)	Validation data (*N* = 50)	Test data (*N* = 50)	*R*²
Prediction of IOL decentration	0.0170 mm²	0.0156 mm²	0.0151 mm²	2	0.0182 mm²	0.0199 mm²	0.0266 mm²	0.265
Prediction of IOL tilt	0.6754°²	0.9409°²	0.8004°²	5	0.8529 °²	1.1319 °²	1.1278 °²	0.391
Prediction of IOL axial position	0.0415 mm²	0.0432 mm²	0.0405 mm²	2	0.0407 mm²	0.0499 mm²	0.0439 mm²	0.671

All models were trained on the training set and tested on the test set. The validation set was used with the FFNN for backpropagation. On the left side, the performance characteristics of the FFNN are listed in terms of mean squared prediction error for the training data, validation data and test data together with the optimal number of iteration cycles (epochs) during training (performance data refer to this iteration). On the right side, the performance characteristics of the MLRM are listed in terms of mean squared prediction error for the training data, validation data and test data together with the coefficient of determination (*R*²) derived from the training set

**Fig. 1:**
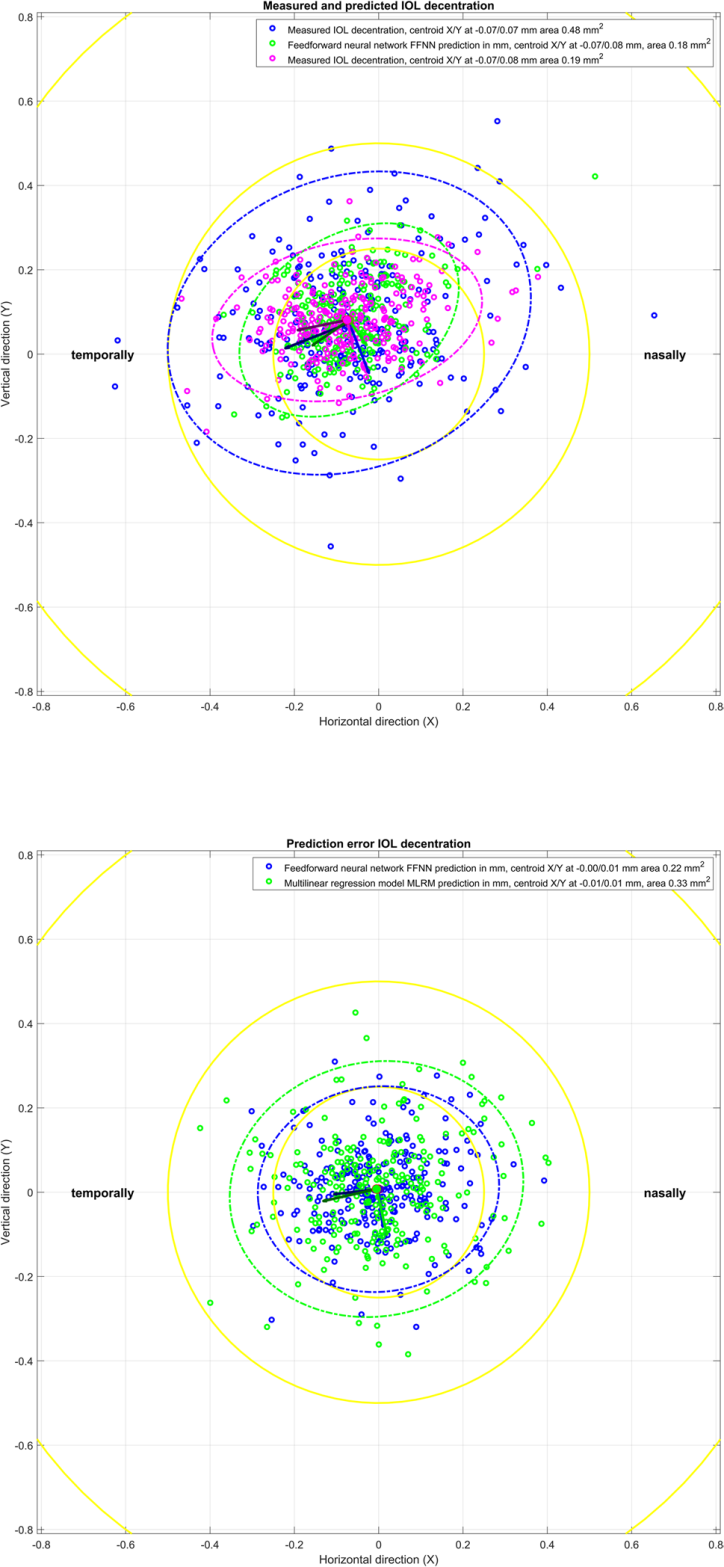
Scatterplot of measured and predicted IOL decentration (upper graph) and scatterplot of prediction error for IOL decentration (lower graph). The *X*/*Y* axis refers to a decentration in the horizontal direction (LDEC_po_X)/vertical direction (LDEC_po_Y). For left eyes, the *X* components of the decentration are reversed in sign in order to present the data in the same orientation as for right eyes. On average, IOLs are slightly decentred towards the upper temporal quadrant. The error ellipses indicate the 95% confidence intervals, and the plot also indicates the centroids (filled circles, *X*/*Y* coordinates mentioned in the legend) together with the orientation of the ellipses (major and minor half axis indicated by dark and bright lines starting at the centroids). The yellow rings indicate IOL decentration below 0.25 mm, 0.5 mm and 1.0 mm, respectively

Figure 1 shows the scatterplot of measured and predicted IOL decentration (in the upper graph) and the scatterplot of prediction error for IOL decentration (in the lower graph). The *X*/*Y* axis refers to a decentration in the horizontal direction (LDEC_po_X)/vertical direction (LDEC_po_Y). For left eyes, the *X* components of the decentration are reversed in sign in order to present the data in the same orientation as for right eyes. We directly see from the graph that, on average, IOLs are slightly decentred towards the upper temporal quadrant (*X*: −0.07 mm/−0.07 mm/−0.07 mm and *Y*: 0.07 mm/0.08 mm/0.08 mm for the measurement/FFNN/MLRM). The error ellipses indicate the 95% confidence intervals, and the plot also indicates the centroids (filled circles, *X*/*Y* coordinates mentioned in the legend) together with the orientation of the ellipses (major and minor half axis indicated by dark and bright lines starting at the centroids). The area of the error ellipse characterising the 95% CI of the prediction error is slightly larger for the MLRM (0.33 mm²) as compared to the FFNN (0.22 mm²).

The Henze-Zirkler test proves that both prediction errors of IOL decentration (with FFNN and MLRM) do not show normality (significance level p = 0.0015 and 0.0004). According to the nonparametric multivariate sign rank test, there was no significant difference (in terms of prediction error) between the two prediction models for IOL decentration (significance level *p* = 0.5005).

The MLRM for prediction of IOL decentration reads:

$${\displaystyle \begin{array}{c} OD:\left[\begin{array}{c}{ pr edLDEC}_{po}X\\ {}{ pr edLDEC}_{po}Y\end{array}\right]=\left[\begin{array}{ccc}-0.2617& 0.0084& 0.0249\kern0.5em 0.8774\kern0.5em 0.1012\\ {}0.9593& -0.0240& \begin{array}{ccc}-0.0676& 0.0840& 0.6057\end{array}\end{array}\right]\bullet \left[\begin{array}{c}1\\ {} AL\\ {}\begin{array}{c}{LT}_{pr}\\ {}{LDEC}_{pr}X\\ {}{LDEC}_{pr}Y\end{array}\end{array}\right]\\ {} OS:\left[\begin{array}{c}{ pr edLDEC}_{po}X\\ {}{ pr edLDEC}_{po}Y\end{array}\right]=\left[\begin{array}{ccc}0.2617& -0.0084& \begin{array}{ccc}-0.0249& 0.8774& -0.1012\end{array}\\ {}0.9593& -0.0240& \begin{array}{ccc}-0.0676& -0.0840& 0.6057\end{array}\end{array}\right]\bullet \left[\begin{array}{c}1\\ {} AL\\ {}\begin{array}{c}{LT}_{pr}\\ {}{LDEC}_{pr}X\\ {}{LDEC}_{pr}Y\end{array}\end{array}\right]\end{array}}$$

**Fig. 2:**
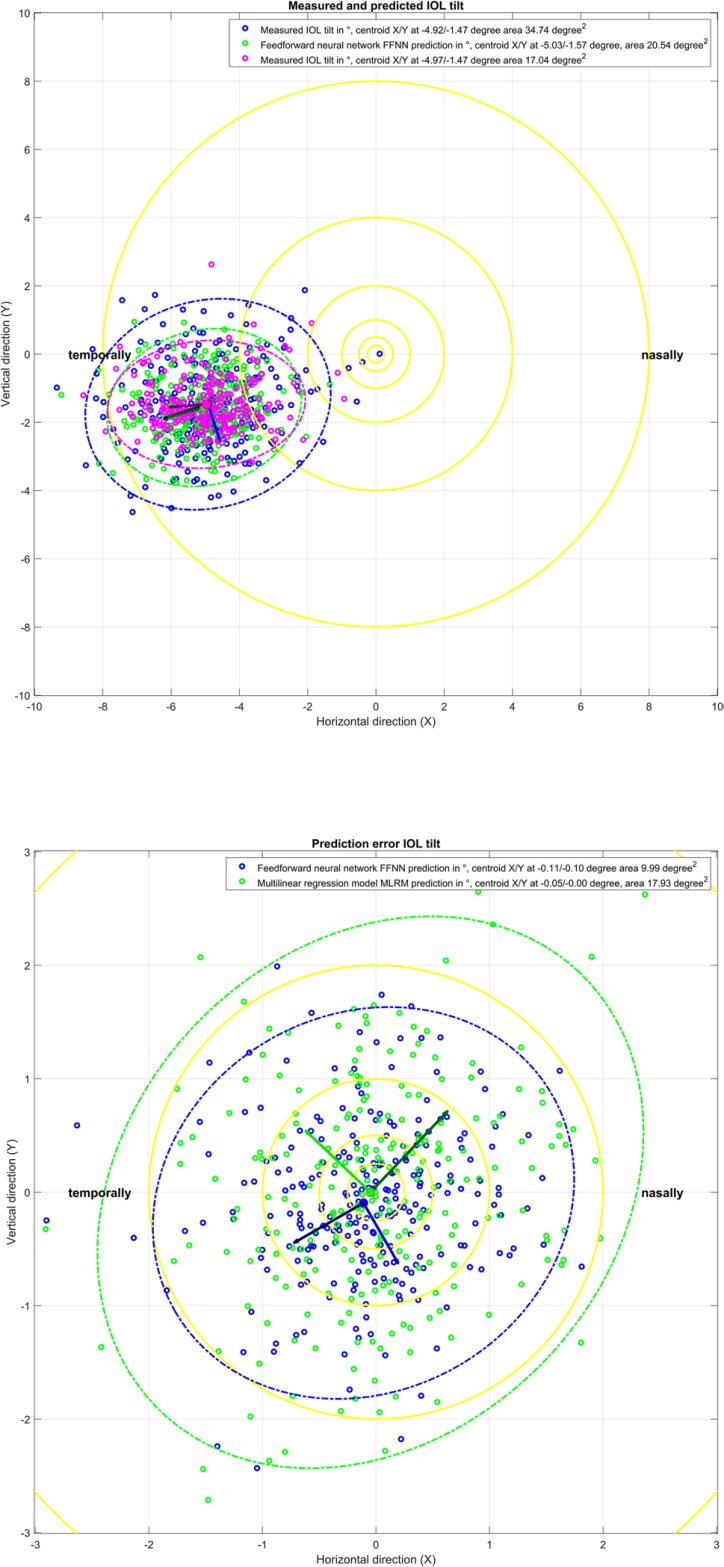
Scatterplot of measured and predicted IOL tilt (upper graph) and scatterplot of prediction error for IOL tilt (lower graph). The *X*/*Y* axis refers to a horizontal tilt (LTILT_po_X)/vertical tilt (LTILT_po_Y) around the *Y*/*X* axis. For left eyes, the *X* components of the tilt are reversed in sign in order to present the data in the same orientation as for right eyes. On average, IOLs are systematically tilted in the temporal direction and slightly tilted in the inferior direction. The error ellipses indicate the 95% confidence intervals, and the plot also indicates the centroids (filled circles, *X*/*Y* coordinates mentioned in the legend) together with the orientation of the ellipses (major and minor half axis indicated by dark and bright lines starting at the centroids). The yellow rings indicate a tilt below 0.25°, 0.5°, 1.0°, 2.0°, 4.0° and 8.0°, respectively

Figure 2 shows the respective scatterplot of measured and predicted IOL tilt (upper graph) and scatterplot of prediction error for IOL tilt (lower graph). The *X*/*Y* axis refers to a horizontal tilt (LTILT_po_X)/vertical tilt (LTILT_po_Y) around the *Y*/*X* axis. Again, for left eyes, the *X* components of the tilt are reversed in sign in order to present the data in the same orientation as for right eyes. On average, IOLs are systematically tilted in the temporal direction (by −4.92°/−5.03°/−4.97° for the measurement/FFNN/MLRM) and slightly tilted in the inferior direction (by −1.47°/−1.57°/−1.47° for the measurement/FFNN/MLRM). The area of the error ellipse characterising the 95% CI of the prediction error is slightly larger for the MLRM (17.93 degree²) as compared to the FFNN (9.99 degree²).

The Henze-Zirkler test proves that both prediction errors of IOL tilt (with FFNN and MLRM) do not show normality (significance level *p* = 0.0014 and 0.0005). According to the nonparametric multivariate sign rank test, there was no significant difference (in terms of prediction error) between the two prediction models for IOL tilt (significance level *p* = 0.4619).

The MLRM for prediction of IOL tilt reads:

$${\displaystyle \begin{array}{c} OD:\left[\begin{array}{c}{ pr edLTILT}_{po}X\\ {}{ pr edLTILT}_{po}Y\end{array}\right]=\left[\begin{array}{ccc}-0.7084& -0.0187& \begin{array}{ccc}-0.0192& 1.7220& \begin{array}{ccc}-0.0663& 0.6647& 0.0077\end{array}\end{array}\\ {}-2.6208& 0.0894& \begin{array}{ccc}-0.2461& 0.9502& \begin{array}{ccc}1.6167& -0.1387& 0.3697\end{array}\end{array}\end{array}\right]\bullet \left[\begin{array}{c}1\\ {} AL\\ {}\begin{array}{c}{LT}_{pr}\\ {}{LDEC}_{pr}X\\ {}\begin{array}{c}{LDEC}_{pr}Y\\ {}{LT ILT}_{pr}X\\ {}{LT ILT}_{pr}Y\end{array}\end{array}\end{array}\right]\\ {} OS:\left[\begin{array}{c}{ pr edLTILT}_{po}X\\ {}{ pr edLTILT}_{po}Y\end{array}\right]=\left[\begin{array}{ccc}0.7084& 0.0187& \begin{array}{ccc}0.0192& 1.7220& \begin{array}{ccc}0.0663& 0.6647& -0.0077\end{array}\end{array}\\ {}-2.6208& 0.0894& \begin{array}{ccc}-0.2461& -0.9502& \begin{array}{ccc}1.6167& 0.1387& 0.3697\end{array}\end{array}\end{array}\right]\bullet \left[\begin{array}{c}1\\ {} AL\\ {}\begin{array}{c}{LT}_{pr}\\ {}{LDEC}_{pr}X\\ {}\begin{array}{c}{LDEC}_{pr}Y\\ {}{LT ILT}_{pr}X\\ {}{LT ILT}_{pr}Y\end{array}\end{array}\end{array}\right]\end{array}}$$

**Fig. 3:**
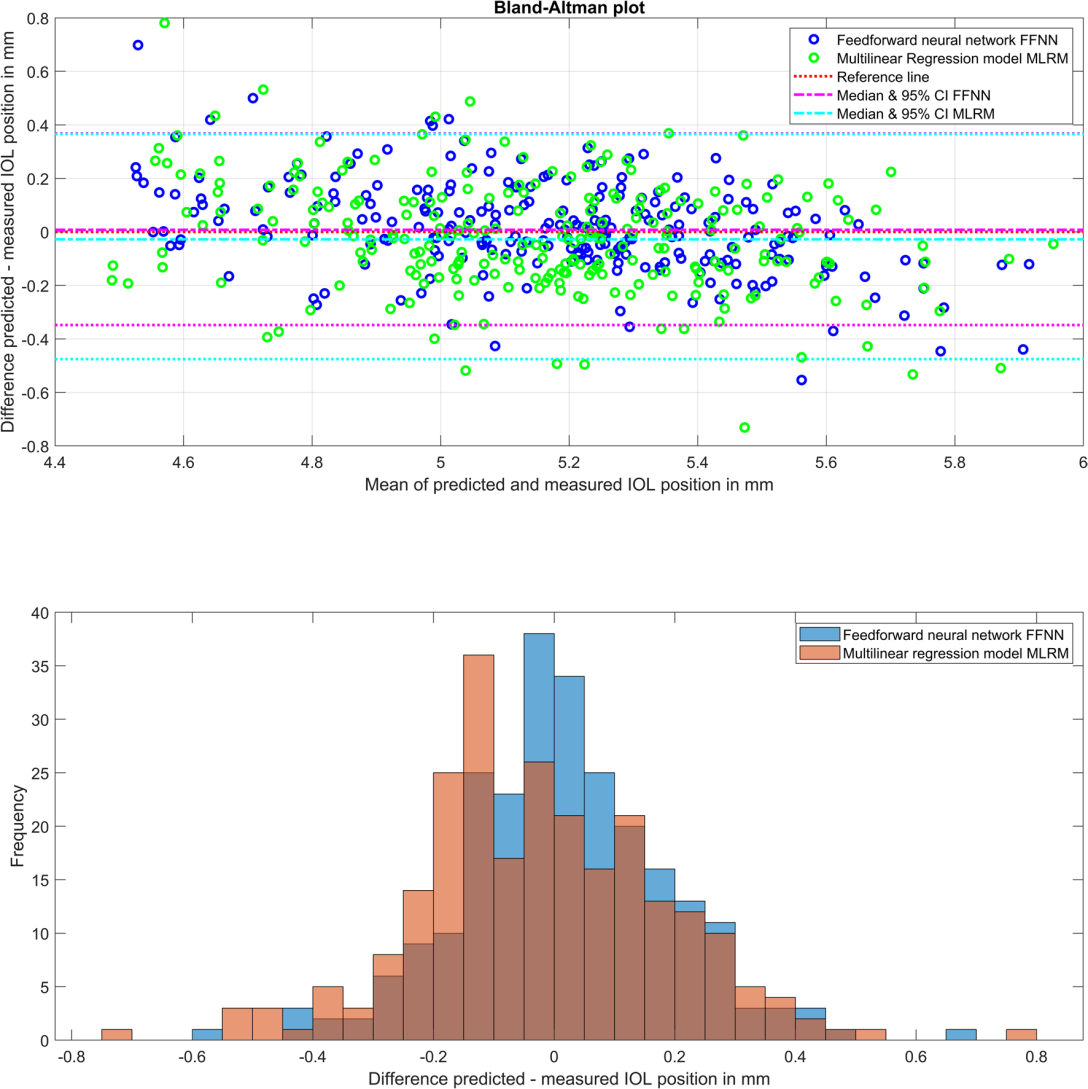
On the upper graph, a Bland-Altman plot shows the predicted vs measured axial IOL position LEQ_po_ in terms of the equator plane. The mean value of measured and predicted LEQ_po_ is shown on the *X* axis, and the difference between predictions from the feedforward neural network FFNN and multilinear regression model MLRM is shown on the *Y* axis together with the reference line (at *Y* = 0) and the medians and 95% confidence intervals (95% CI) for both prediction models. On the lower graph, the distribution (frequency) of the prediction errors (difference predicted – measured LEQ_po_) for the FFNN and MLRM is shown in a histogram plot

Figure 3 displays on the upper graph a Bland-Altman plot showing the predicted vs. measured axial IOL equator plane position LEQ_po_. The mean value of measured and predicted LEQ_po_ is shown on the *X* axis, and the difference between feedforward neural network FFNN and multilinear regression model MLRM prediction is shown on the *Y* axis, together with the reference line (at *Y* = 0) and the medians and 95% confidence intervals (95% CI) for both prediction models. On the lower graph, the distribution of the prediction errors (prediction – measurement of LEQ_po_) for the FFNN and MLRM is shown in a histogram plot. The boundaries of the 95% CI are slightly narrower with the FFNN (magenta dashed lines) compared to the MLRM (cyan dashed lines). From the histograms, we do not see a systematic difference between the distributions of the prediction errors with the FFNN and the MLRM.

The Shapiro-Wilk test proves that both prediction errors of axial IOL equator plane position (with FFNN and MLRM) show normality (significance level *p* = 0.0536 and 0.0775). According to the parametric *t*-test, the FFNN prediction model for the axial IOL position showed significantly better performance (in terms of prediction error) compared to the MLRM (significance level *p* < 0.0001).

The MLRM for prediction of the axial equator position of the IOL reads:

$${\displaystyle \begin{array}{ccc} OD:\left[p{redLEQ}_{po}\right]& =& \left[\begin{array}{ccc}1.6122& 0.0346& \begin{array}{cc}0.5799& 0.4158\end{array}\end{array}\right]\bullet \left[\begin{array}{c}1\\ {} AL\\ {}\begin{array}{c}{LEQ}_{pr}\\ {}{LDEC}_{pr}X\end{array}\end{array}\right]\\ {} OS:\left[p{redLEQ}_{po}\right]& =& \left[\begin{array}{ccc}1.6122& 0.0346& \begin{array}{cc}0.5799& -0.4158\end{array}\end{array}\right]\bullet \left[\begin{array}{c}1\\ {} AL\\ {}\begin{array}{c}{LEQ}_{pr}\\ {}{LDEC}_{pr}X\end{array}\end{array}\right]\end{array}}$$

## Discussion

Prediction of the pseudophakic lens position is one of the major challenges in biometry and IOL power calculation before cataract surgery [5, 29, 30]. In general, the term ‘pseudophakic lens position’ is not well defined. In the literature, it is used either as the effective lens position ‘ELP’ as a fictitious parameter denoting the position a thin lens with its labelled power has to be placed in the eye to obtain the required refraction at the spectacle plane or to refer to the real geometric (or anatomical) axial lens position ‘ALP’ as measured postoperatively with modern optical tomographers or biometers [12]. We must, however, be aware that there is no common standard as to whether the axial position of the front apex, back apex, IOL equator or the image side principal plane is mentioned. Since the ELP is a fictitious parameter and is biassed by all assumptions and simplifications in IOL power calculation schemes based on a pseudophakic model eye, this value cannot be measured by any practical means [5].

However, in general, the term pseudophakic lens position is not restricted to the axial position in terms of ELP or ALP. In addition to the axial position, there are 5 more degrees of freedom in terms of lateral displacement or decentration in *X* and *Y*, tilt in *X* and *Y* (around *Y* and *X*) and IOL rotation around *Z*. In rotationally symmetric lenses, the axial alignment (rotation around *Z*) is not relevant, but as soon as we are concerned with toric or sectorial lenses, the axis orientation is of high relevance. IOL decentration and tilt typically have a minor impact on the spectacle refraction after cataract surgery, but both could significantly affect the visual performance of the eye by inducing aberrations of higher order (e.g. coma) [2, 3, 7, 9, 11, 13]. As such aberrations cannot be fully corrected by glasses or contact lenses, they may induce some discomfort or be considered to be disturbing by the patient.

Proper positioning of the lens in the eye with respect to the visual axis has a high impact on visual performance, especially in the segment of premium lenses [14]. It is well known from the literature that aberration-correcting aspheric lens designs can induce photic phenomena if decentred or tilted, and even more so for refractive or diffractive bifocal or multifocal or EDOF lenses. Patients could be disappointed even if the surgery was otherwise uneventful [6, 8].

Modern OCT techniques, as established in ophthalmology a decade ago, are capable of measuring the anterior eye segment including cornea and lens [12]. In addition to the segmentation of the cornea with its front and back surface, as already available with Scheimpflug tomographers, some software tools in the OCT application also allow for automatic segmentation of the crystalline lens or IOL front and back surface (lens analysis module). However, as with all optical measurement techniques, the iris pigment blocks the light and proper segmentation of the lens or IOL surfaces requires a sufficient pupil size [14]. In the lens analysis module of the Casia2, the crystalline lens boundaries are automatically detected and fitted by spherical surface models, enabling estimation of the radii of curvature of both surfaces, the diameter of the lens equator, the extraction of the equator plane LEQ_pr_ (and therefore the split of the crystalline lens in its anterior and posterior segment) and analysis of lens decentration and tilt. After cataract surgery, the capabilities of this lens analysis module are restricted to a measurement of the IOL tilt and decentration and detection of the IOL front and back apex. Therefore, the IOL equator cannot be directly assessed, and we decided to define the equator plane of the IOL LEQ_po_ as half the distance between the front and back apex, assuming an equiconvex IOL design. If the exact IOL design is known, our model could easily be refined to predict the ‘real’ equator plane instead [28, 29].

In the present study, in addition to biometric data from the IOLM, we extracted measurements of the cornea and lens before and after cataract surgery from the CASIA. Assuming lateral symmetry between left and right eyes, the *X* components of the decentration were reversed for left eyes in sign in order to present the data in the same orientation as for right eyes. We subsequently used a stepwise linear regression method [15] to identify the relevant predictors for our prediction models in order to estimate the IOL decentration and tilt as well as the axial IOL equator position. As decentration and tilt are both vector metrics as provided from the CASIA in terms of vector magnitude and orientation angle, we converted the data to *X* and *Y* components and set up a multivariate prediction model which also includes the interaction between the *X* and *Y* components. For the prediction of the axial IOL equator plane position (as a scalar parameter), a simple univariate model is sufficient. We implemented 2 different models for each prediction: First, we set up a shallow feedforward neural network [20, 30], and second, we implemented a multilinear regression model (bivariate for decentration and tilt and univariate for axial IOL equator position) as a reference [23].

We found that all 6 models (FFNN and MLRM for predicting IOL decentration, tilt and axial position) perform quite well. Our results indicate that the prediction of IOL decentration shows, in general, the lowest performance (e.g. *R*² = 0.265 for the MLRM), whereas the prediction of IOL tilt (*R*² = 0.391) and especially the prediction of the axial IOL equator position (*R*² = 0.64) both show higher performance. This means that using biometric data from the IOLM and preoperative measures from the CASIA including the data extracted from the lens analysis module, we could make a statistical prediction of the lens decentration and tilt and especially of the axial IOL equator position. We argue that a prediction of the ‘real’ equator position based on the design data of the IOL would be expected to have a similar performance compared to the prediction of LEQ_po_ that we used in this study.

What we directly understand from these results is that the data scatter of lateral IOL decentration in *X* and *Y* does not show a systematic direction. Although the centroid shown in Fig. 1 in the upper graph is slightly shifted towards the upper temporal quadrant, there is a large scatter of the data, as indicated by the 95% error ellipses. Interestingly, the error ellipses (major half axes) are oriented mainly in the 1st and 3rd quadrants, indicating that the variation is largest more or less perpendicular to the centroid offset. The prediction error shown in the lower graph shows no trend error (centroids close to *X* = *Y* = 0) and no systematic orientation of the error ellipses (the sizes of the respective major and minor half axes are comparable). The area of the error ellipse for the fit error shown in the legend indicates that the FFNN performs slightly better as compared to the MLRM (0.22 mm² vs 0.33 mm²), but without statistical significance (result of the multivariate nonparametric sign rank test). The upper graph in Fig. 2 shows a systematic offset of the IOL tilt *X* and *Y* components from zero with respect to the instrument axis of the CASIA. The centroids are located at a tilt of around 5° in the temporal direction and 1.5° in the inferior direction. The temporal tilt coincides mostly with the tilt of the entrance beam in the Liou-Brennan schematic model eye (angle alpha or kappa), which is due to the temporal shift of the fovea relative to the posterior pole of the eye. However, the inferior shift of IOL tilt is not well reflected by any schematic model eye. The prediction error shown in the lower graph again shows no trend error (centroids close to *X* = *Y* = 0), but a slight orientation of the error ellipses (sizes of the respective major and minor half axes) towards the main tilt direction. The area of the error ellipse for the fit error shown in the legend indicates that the FFNN performs slightly better as compared to the MLRM (9.99 vs. 17.93 degree²), but again without statistical significance (result of the multivariate nonparametric sign rank test). From the Bland-Altman plot in Fig. 3, we learn that even though the prediction of LEQ_po_ showed the best overall performance, the variation according to the 95% confidence interval (upper graph) is around ±0.3 mm. For an average eye, a variation of ±0.3 mm in the axial IOL position refers to a shift in spectacle refraction of around ±0.45 dpt. Again, the FFNN prediction shows a slightly better performance, as indicated by the narrower 95% confidence interval, and the distribution shown in the histogram in the lower graph is slightly less tailed compared to the MLRM. The Student’s *t*-test shows that the slight differences in performance of both prediction models are statistically significant.

There are, however, some limitations of our study: Firstly, we used a dataset where 4 different lens types were used during cataract surgery and the number of eyes considered in this study is not high. Therefore, this paper has the character of a ‘pilot study’, and with larger datasets, a subgroup analysis could bring out the differences in IOL decentration, tilt and equator position between different lens types and materials. Secondly, as the exact design data of the lenses are not disclosed, we defined the middle plane between the IOL front and back apex as the equator plane. This is valid for equiconvex (or equiconcave) IOL designs but might give imprecise predictions for highly asymmetric IOL designs. Last but not least, we used the decentration and tilt data from the lens analysis module of the CASIA. Since all measurements were performed with fixation of the internal target, we assume that all data are referenced to the instrument axis/fixation axis of the device during measurement. However, in the CASIA operating manual, there is no detailed information on the reference of the lens or IOL decentration or tilt.

In conclusion, this study provides modern neural network-based and classical multilinear regression-based predictions for intraocular lens lateral decentration, IOL tilt and axial IOL equator position based on biometric data and preoperative measures from the Casia2 anterior segment tomographer. Prediction of IOL decentration generally showed the poorest performance with a data scatter of around 0.3 mm, whereas prediction of IOL tilt showed a better performance with a data scatter of around 1.5°. The prediction of the axial position of the IOL equator plane showed the best performance with a data scatter of around ±0.3 mm. The neural network approach, in general, showed slightly superior results compared to the classical multilinear regression, but with statistically significant differences only for the prediction of the axial IOL equator position.
